# Trends of HIV/Syphilis/HSV-2 seropositive rate and factors associated with HSV-2 infection in men who have sex with men in Shenzhen, China: A retrospective study

**DOI:** 10.1371/journal.pone.0251929

**Published:** 2021-05-20

**Authors:** Sha-Sha Mao, Shui-Dong Feng, Chen-Li Zheng, Wei Hu, Hao Li, Jie Tang, Zheng-Rong Yang, Jin Zhao

**Affiliations:** 1 School of Public Health, University of South China, Hengyang, Hunan, China; 2 Department of HIV/AIDS Control and Prevention, Shenzhen Center for Disease Control and Prevention, Shenzhen, Guangdong, China; 3 Shenzhen Center for Chronic Disease Control, Shenzhen, Guangdong, China; China Medical University, CHINA

## Abstract

**Objectives:**

To analyze the trends of HIV/syphilis/HSV-2 seropositive rate and explore the related factors with HSV-2 infection to provide the basis for adjusting STD intervention strategies and formulating prevention and control measures among MSM in Shenzhen.

**Methods:**

Time-location sampling was conducted among MSM in Shenzhen in 2012, 2014, 2016, and 2018. Data on demographics, sexual behaviors and the laboratory test results of HIV, syphilis, HSV-2 were collected. The χ^2^ trend test was used to analyze the trends of HIV/syphilis/HSV-2 seropositive rate. The binary logistic regression model was used to explore the factors associated with HSV-2 infection.

**Results:**

The seropositive rate of HIV fell significantly from 15.9% in 2012 to 8.7% in 2018 (*P*_*trend*_ = 0.003), syphilis seropositive rate was significantly decreased from 20.4% in 2012 to 14.8% in 2018 (*P*_*trend*_ = 0.025), HSV-2 seropositive rate had no significant change (16.7% in 2012 to 14.0% in 2018; *P*_*trend*_ = 0.617). In principal component logistic regression analysis showed that FAC1_1 (X_1_ = Ever had sex with female, X_2_ = Gender of first sexual partner, X_3_ = Marital status, X_4_ = Age group), FAC2_1 (X_5_ = Education, X_6_ = Monthly income (RMB), X_7_ = Frequency of condom use in anal sex with men in the past 6 months), and FAC4_1 (X_9_ = History of STDs) were significantly associated with HSV-2 infection.

**Conclusions:**

The seropositive rates of HIV and syphilis have dropped significantly but are still high. HSV-2 seropositive rate had no significant change and maintained a high level. It is necessary to continue strengthening HIV and syphilis interventions among MSM in Shenzhen. HSV-2 detection and intervention are urgently required for MSM, which might be another effective biological strategy further to control the HIV epidemic among MSM in Shenzhen.

## Introduction

The HIV epidemic is worsening among men who have sex with men (MSM) [1]. It is imperative to explore more effective methods that could curb the HIV epidemic in this population. Sexually transmitted diseases (STDs) such as syphilis and herpes simplex virus type 2 (HSV-2) are biological risk factors for HIV infection and transmission [2,3]. Therefore, it is essential to carry out STDs routine surveillance and intervention for MSM. In the past decade, the routine surveillance and intervention of STDs for MSM in China have focused on HIV, syphilis and Hepatitis C (HCV). In contrast, HSV-2 detection and intervention have not got much attention.

HSV-2 infection is the leading cause of genital ulcer disease, one of the most common sexually transmitted diseases in the world [4]. MSM have high rates of HSV-2 infection, ranging from 8.7% to 65% in studies of different countries, especially in HIV positive MSM, which was more severe, ranging from 20.4% to 55.9% [5–9]. HSV-2 has a strong interaction with HIV, which can increase the risk of acquiring HIV by 2–4 fold and transmitting HIV by 2–5 fold, even promote the progression of HIV disease [3,10,11].

Most people infected with HSV-2 are asymptomatic but have virus shedding in the anogenital sites. Consequently, subclinical virus shedding of unrecognized and undiagnosed appear to be major factors in the spread of HSV-2 [12]. HSV-2 serum-specific antibody detection is of great significance in the early recognition and diagnosis of HSV-2 infection, particularly for atypical genital ulcer disease and asymptomatic carriers [13]. Currently, therapy of HSV-2 with acyclovir, famciclovir, or valacyclovir can reduce the recurrence of genital ulcers and the frequency of HSV-2 shedding. It can even decrease the level of HIV RNA in plasma and genital secretions, which may bring more clinical and public health benefits for patients with HIV-1 and HSV-2 coinfection [14].

Therefore, the detection and intervention of HSV-2 can be used as a biological strategy to control HIV infection and transmission, which may have a profound impact on the global HIV epidemic. Moreover, HSV-2 and HIV prevalence are strongly associated. HSV-2 prevalence can be used as a proxy ‘biomarker’ of HIV epidemic potential, acting as a ‘temperature scale’ of the intensity of sexual risk behavior that is difficult to measure directly [15]. Given these benefits, HSV-2 detection and intervention for MSM can track the intensity of sexual behavior, predict the epidemic potential of HIV, and even help control the HIV epidemic in MSM.

With the continuous strengthening of the prevention and control of HIV and syphilis among MSM in Shenzhen, the prevalence of HIV and syphilis has decreased but is still higher than other population, notably for MSM recruited from gay venues (i.e., gay bars, saunas). It is necessary to explore another effective and practical routine detection index to further control the HIV epidemic among MSM in Shenzhen. However, HSV-2 detection and intervention have never conducted for MSM in Shenzhen. Therefore, this study aims to access the trends of HIV, syphilis, and HSV-2 seropositive rate and explore the related factors with HSV-2 infection to provide the basis for adjusting STD intervention strategies and formulating prevention and control measures among MSM in Shenzhen.

## Materials and methods

### Data collection

Four years of cross-sectional data (questionnaire and laboratory test results) was selected from Shenzhen Center for Disease Control and Prevention (Shenzhen CDC) in China. Surveys have been conducted by Shenzhen CDC and 258 Rainbow Working Group (Made up of MSM volunteers) using the time-location sampling method and covered all MSM-frequented venues (e.g. bars, saunas, recreational and massage centers). Inclusion criteria for MSM: 1) biologically male, 2) self-reported having sex with men (anal or oral sex) in the previous 6 months, 3) willing to participate in the survey. Before the survey commenced, eligible participants had to sign a consent form, and then they were invited to complete a questionnaire that included sociodemographic and sexual behavior characteristics (shown in Table 1). After the questionnaire interview, the blood sample was obtained from the subject for laboratory testing. A unique participant ID was used to link the questionnaire information and the laboratory test results. The study was approved by the Internal Review Board of the Shenzhen Center for Disease Control and Prevention (Approval No 2019-060A).

**Table 1:** Demographic and behavioral characteristics among MSM in Shenzhen, China (2012–2018).

Characteristic	Overall *N* = 1695	2012 n = 496	2014 n = 457	2016 n = 398	2018 n = 344	*P*
**Age (`X±S)**						0.549^a^
	31.5±8.38	31.1±8.16	31.7±8.44	31.7±8.32	31.8±8.69	
**Age group**						0.289
≤24	322 (19.0)	106 (21.4)	87 (19.0)	67 (16.8)	62 (18.0)	
25–34	867 (51.2)	254 (51.2)	233 (51.0)	203 (51.0)	177 (51.5)	
35–44	374 (22.1)	101 (20.4)	107 (23.4)	98 (24.6)	68 (19.8)	
≥45	132 (7.8)	35 (7.1)	30 (6.6)	30 (7.5)	37 (10.8)	
**Residence permit**						**<0.001**
Shenzhen	297 (17.5)	50 (10.1)	86 (18.8)	87 (21.9)	74 (21.5)	
Other cities of Guangdong Province	293 (17.3)	74 (14.9)	88 (19.3)	59 (14.8)	72 (20.9)	
Other Provinces	1105 (65.2)	372 (75.0)	283 (61.9)	252 (63.3)	198 (57.6)	
**Residence in Shenzhen (years)**						0.663
0	107 (6.3)	28 (5.6)	28 (6.1)	33 (8.3)	18 (5.2)	
<1	290 (17.1)	88 (17.7)	77 (16.8)	70 (17.6)	55 (16.0)	
1–2	278 (16.4)	90 (18.1)	73 (16.0)	56 (14.1)	59 (17.2)	
>2	1020 (60.2)	290 (58.5)	279 (61.1)	239 (60.1)	212 (61.6)	
**Education**						0.125
Middle school or less	344 (20.3)	110 (22.2)	95 (20.8)	74 (18.6)	65 (18.9)	
High school	534 (31.5)	164 (33.1)	155 (33.9)	121 (30.4)	94 (27.3)	
college and above	817 (48.2)	222 (44.8)	207 (45.3)	203 (51.0)	185 (53.8)	
**Employment**						**0.001**
Full-time employed	1450 (85.5)	454 (91.5)	378 (82.7)	324 (81.4)	294 (85.5)	
Part-time /self-employed	104 (6.1)	17 (3.4)	37 (8.1)	29 (7.3)	21 (6.1)	
Unemployed/retired/student/other	141 (8.3)	25 (5.0)	42 (9.2)	45 (11.3)	29 (8.4)	
**Monthly income (RMB)**						**<0.001**
≤3000	392 (23.1)	182 (36.7)	107 (23.4)	64 (16.1)	39 (11.3)	
3001–5000	703 (41.5)	223 (45.0)	223 (48.8)	160 (40.2)	97 (28.2)	
>5000	600 (35.4)	91 (18.3)	127 (27.8)	174 (43.7)	208 (60.5)	
**Marital status**						**0.004**
Unmarried	1282 (75.6)	390 (78.6)	327 (71.6)	304 (76.4)	261 (75.9)	
Married/cohabiting	285 (16.8)	80 (16.1)	99 (21.7)	58 (14.6)	48 (14.0)	
Widowed/separated/divorced	128 (7.6)	26 (5.2)	31 (6.8)	36 (9.0)	35 (10.2)	
**Sexual orientation**						**<0.001**
Homosexual	1189 (70.1)	342 (69.0)	308 (67.4)	284 (71.4)	255 (74.1)	
Bisexual	373 (22.0)	134 (27.0)	103 (22.5)	73 (18.3)	63 (18.3)	
Heterosexual or unsure	133 (7.8)	20 (4.0)	46 (10.1)	41 (10.3)	26 (7.6)	
**Age at sex debut**						0.052
≤20	649 (38.3)	214 (43.1)	159 (34.8)	149 (37.4)	127 (36.9)	
>20	1046 (61.7)	282 (56.9)	298 (65.2)	249 (62.6)	217 (63.1)	
**Ever had sex with female**						0.069
No	934 (55.1)	285 (57.5)	233 (51.0)	234 (58.8)	182 (52.9)	
Yes	761 (44.9)	211 (42.5)	224 (49.0)	164 (41.2)	162 (47.1)	
**Gender of first sexual partner**						0.336
Male	1184 (69.9)	344 (69.4)	308 (67.4)	291 (73.1)	241 (70.1)	
Female	511 (30.1)	152 (30.6)	149 (32.6)	107 (26.9)	103 (29.9)	
**Gender of sexual partner in P6M**						0.394
Male	1517 (89.5)	436 (87.9)	407 (89.1)	360 (90.5)	314 (91.3)	
Male and female	178 (10.5)	60 (12.1)	50 (10.9)	38 (9.5)	30 (8.7)	
**Number of male sex partners in P6M**						**<0.001**
1	414 (24.4)	89 (17.9)	106 (23.2)	111 (27.9)	108 (31.4)	
2–4	731 (43.1)	224 (45.2)	198 (43.3)	157 (39.4)	152 (44.2)	
≥ 5	318 (18.8)	155 (31.3)	72 (15.8)	46 (11.6)	45 (13.1)	
**Frequency of condom use in anal sex with men in P6M**						**<0.001**
Never	390 (23.0)	310 (62.5)	27 (5.9)	29 (7.3)	24 (7.0)	
Sometimes (<50%)	262 (15.5)	84 (16.9)	81 (17.7)	51 (12.8)	46 (13.4)	
Always (>50%)	253 (14.9)	28 (5.6)	100 (21.9)	65 (16.3)	60 (17.4)	
Every time	561 (33.1)	54 (10.9)	165 (36.1)	169 (42.5)	173 (50.3)	
**Had STD-related symptoms in the past year**						0.218
No	1417 (83.6)	428 (86.3)	382 (83.6)	327 (82.2)	280 (81.4)	
Yes	278 (16.4)	68 (13.7)	75 (16.4)	71 (17.8)	64 (18.6)	
**History of STDs** ^ψ^						0.275
No	1557 (91.9)	464 (93.5)	420 (91.9)	364 (91.5)	309 (89.8)	
Yes	138 (8.1)	32 (6.5)	37 (8.1)	34 (8.5)	35 (10.2)	
**HIV infection**						0.003^b^
No	1449 (85.5)	417 (84.1)	377 (82.5)	341 (85.7)	314 (91.3)	
Yes	246 (14.5)	79 (15.9)	80 (17.5)	57 (14.3)	30 (8.7)	
**Syphilis infection**						0.025^b^
No	1400 (82.6)	395 (79.6)	377 (82.5)	335 (84.2)	293 (85.2)	
Yes	295 (17.4)	101 (20.4)	80 (17.5)	63 (15.8)	51 (14.8)	
**HSV-2 infection**						0.617^b^
No	1427 (84.2)	413 (83.3)	392 (85.8)	326 (81.9)	296 (86.0)	
Yes	268 (15.8)	83 (16.7)	65 (14.2)	72 (18.1)	48 (14.0)	

NOTE.

**Abbreviations:** MSM, men who have sex with men; RMB, Renminbi; P6M, in the past 6 months; STDs, sexually transmitted diseases.

^※^ ‘symptoms’ here including at least one of the following: Painful urination or burning sensation, Abnormal of urethral discharge, Genital ulcers or warts, Anal ulcers or secretions, etc.

^ψ^ ‘STDs’ here including at least one of the following: Condyloma acuminate, gonorrhea, urethritis, Chlamydial infection, Hepatitis B, etc. (except for HIV/syphilis infection).

^a^ *P*: Calculated by analysis of variance (ANOVA)

^b^ *P* = *P*_*trend*_.

Statistically significant variables (*P* <0.05) are shown in boldface.

### Laboratory testing

HIV was tested by using a rapid test (Determine HIV-1/2/O, Abbott Laboratories, IL) and enzyme-linked immunosorbent (ELISA) assay (Wantai Biotech Inc., Beijing) for screening and western blot (WB) analysis (Genlabs Diagnostics, Singapore) for confirmation. Syphilis was tested with ELISA (Wantai Biotech Inc, Beijing, China) for screening and Treponema pallidum particle agglutination (TPPA) assay (Fujirebio Inc, Japan) for confirmation. HSV-2 serum antibodies were detected using specific IgG2 ELISA kits (Beier Company, Beijing). All tests were performed in the laboratory of Shenzhen Centre for Disease Control and Prevention (CDC), China and according to the manufacturers’ instructions.

### Statistical analysis

All analyses were performed using SPSS 24.0 (SPSS Inc., Chicago, IL). Chi-square test and analysis of variance (ANOVA) were conducted to compare demographic and behavioral characteristics in different years. The χ^2^ trend test was used to estimate the trends of HIV/HSV-2/syphilis seropositive rate and further evaluate the age-specific trends of HIV/HSV-2/syphilis. The binary logistic regression model was used to explore the factors associated with HSV-2 infection. Univariable analysis results were expressed by the crude odds ratio (COR) and 95% confidence intervals (95% CIs). Condition index and variance decomposition proportion were used to test the multicollinearity between variables. The condition index≥10 or 30 and variance decomposition proportion >0.5 can be regarded as obviously collinear. The principal component analysis (PCA) was used to eliminate the collinearity. The extracted factors by the principal component analysis were included in the multivariate analysis, and the adjusted ORs (AOR) and 95% CIs were presented. All statistical tests were two-sided, and *P*-value < 0.05 was considered statistically significant.

## Results

### 1. Demographic and behavioral characteristics among MSM in Shenzhen, China (2012–2018)

**Fig 1:**
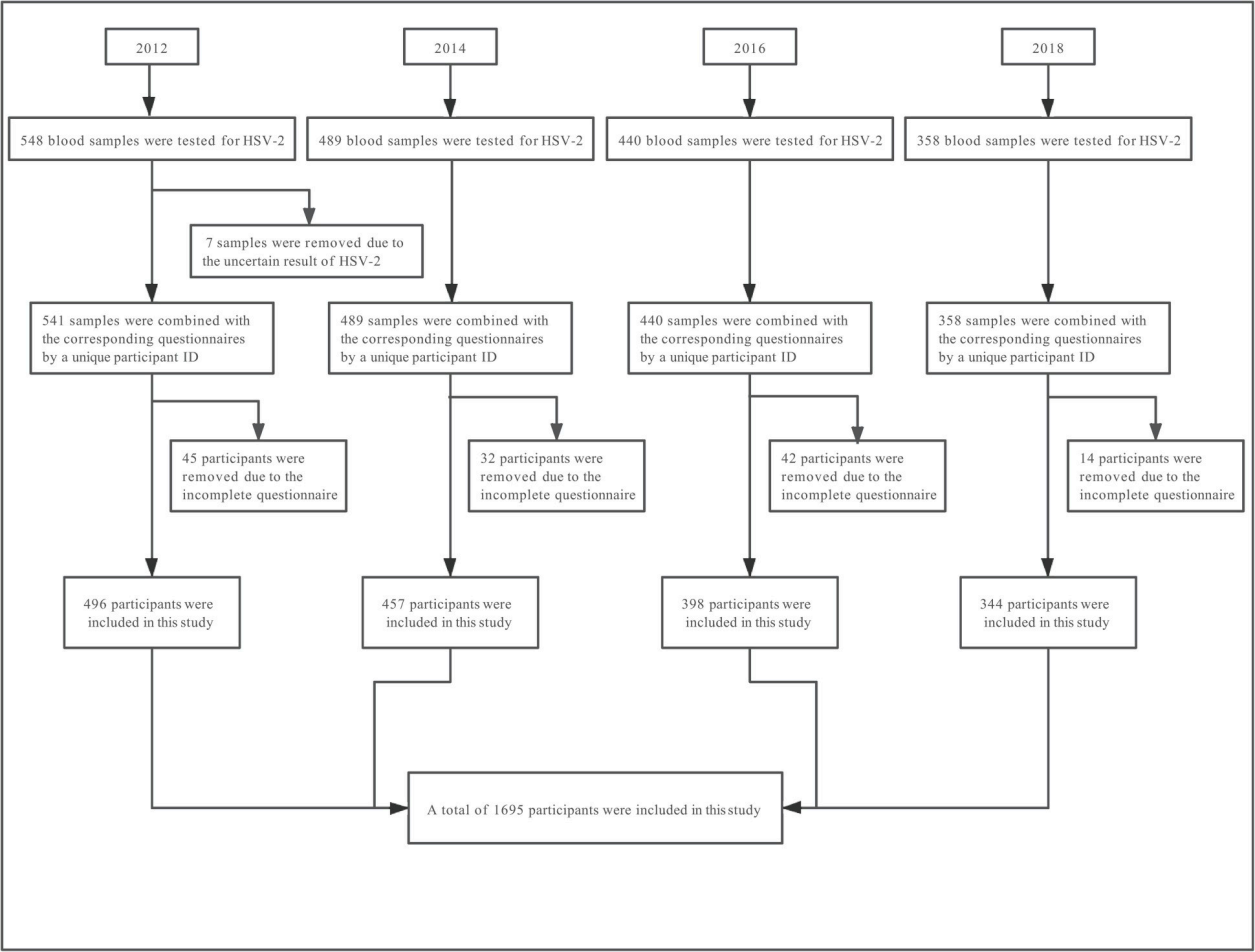
Flow chart of participant selection in Shenzhen, China. A total of 1835 samples were tested for HSV-2, and 140 were removed because of uncertain result or incomplete questionnaire.

A total of 1695 participants, including 2012(n = 496), 2014(n = 457), 2016(n = 398), 2018(n = 344), the flow chart of participant selection is shown in Fig 1. Among all participants, the average age of cases was 31.5±8.38 years old, more than half (51.2%) were 25–34 years old, and only 7.8% were 45 years old and above. 82.5% of them did not have permanent residency in Shenzhen, and 60.2% lived in this city for more than two years. 48.2% of participants had a college degree and above. In terms of occupation, 85.5% of MSM were full-time employed, and 35.4% had a monthly income>5000 RMB. 75.6% of MSM were unmarried, and 70.1% were self-identified as homosexual. 61.7% of cases had sex debut after 20 years old. 44.9% of participants ever had sex with female. 69.9% of MSM experienced sexual debut with male, 30.1% with female, respectively. 89.5% of MSM only had homosexual behaviors in the past 6 months, while 10.5% experienced bisexual behaviors. 43.1% of MSM had 2 to 4 male sex partners in the past 6 months. 23.0% of participants never used condom in anal sex with men in the past 6 months, whereas only 33.1% used condom continuously. 8.1% of participants had sexually transmitted diseases (STDs), and 16.4% reported they had one or more STD-related symptoms in the past year. The distribution of these variables in different years was significantly different: residence permit, employment, monthly income, marital status, sexual orientation, number of male sex partners in P6M, frequency of condom use in anal sex with men in P6M (Table 1).

### 2. Trends analysis of HIV/HSV-2/syphilis seropositive rate among MSM in Shenzhen, China (2012–2018)

**Fig 2:**
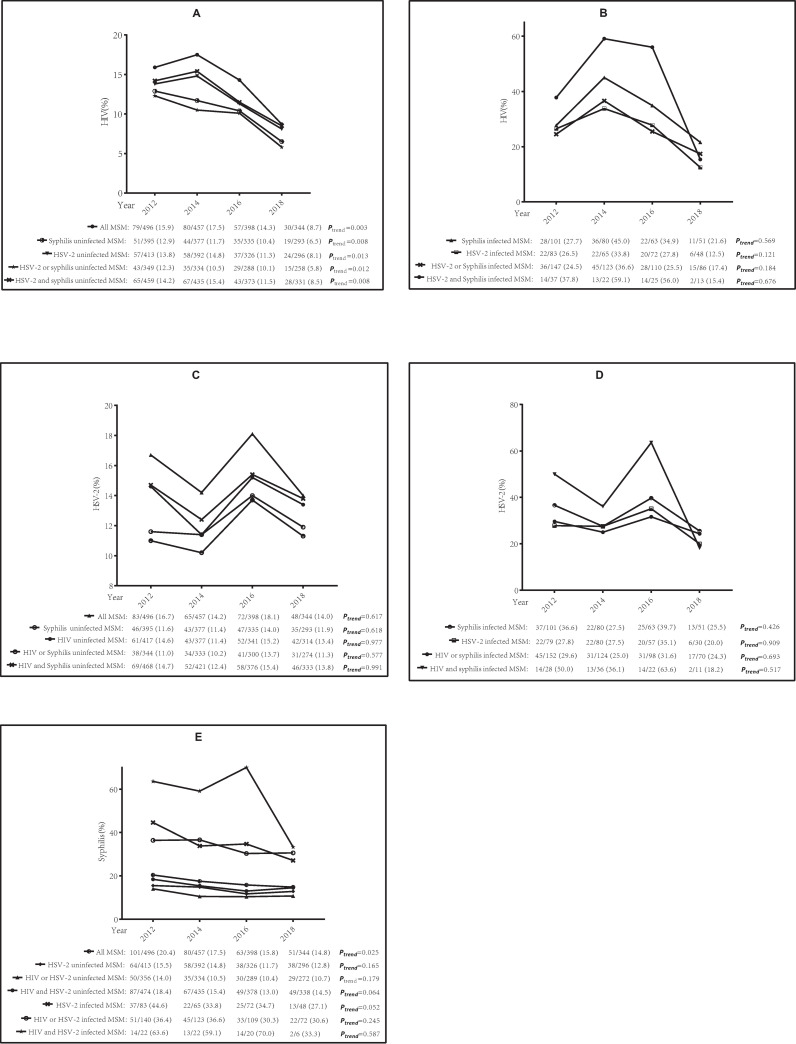
Trends analysis of HIV/HSV-2/syphilis seropositive rate among MSM in Shenzhen, China (2012–2018). (A) Trend analysis of HIV seropositive rate among all MSM and subgroups (Syphilis uninfected MSM, HSV-2 uninfected MSM, HSV-2 or syphilis uninfected MSM, HSV-2 and syphilis uninfected MSM). (B) Trend analysis of HIV seropositive rate among subgroups (Syphilis infected MSM, HSV-2 infected MSM, HSV-2 or Syphilis infected MSM, HSV-2 and Syphilis infected MSM). (C) Trend analysis of HSV-2 seropositive rate among all MSM and subgroups (Syphilis uninfected MSM, HIV uninfected MSM, HIV or Syphilis uninfected MSM, HIV and Syphilis uninfected MSM). (D) Trend analysis of HSV-2 seropositive rate among subgroups (Syphilis infected MSM, HSV-2 infected MSM, HIV or syphilis infected MSM, HIV and syphilis infected MSM). (E) Trend analysis of syphilis seropositive rate among all MSM and subgroups (HSV-2 uninfected MSM, HIV or HSV-2 uninfected MSM, HIV and HSV-2 uninfected MSM, HSV-2 infected MSM, HIV or HSV-2 infected MSM, HIV and HSV-2 infected MSM).

The testing yield of HIV in all MSM decreased significantly from 2012 to 2018 (15.9% to 8.7%; *P*_*trend*_ = 0.003) and the reductions were consistent in subgroup analyses: syphilis uninfected MSM (12.9% to 6.5%; *P*_*trend*_ = 0.008), HSV-2 uninfected MSM (13.8% to 8.1%; *P*_*trend*_ = 0.013) (Fig 2A and 2B). HSV-2 seropositive rate had no significant change among all MSM (16.7% to 14.0%; *P*_*trend*_ = 0.617) and subgroups (Fig 2C and 2D). A significantly decreasing trend was identified of syphilis seropositive rate from 2012 to 2018 in all MSM (20.4% to 14.8%; *P*_*trend*_ = 0.025), but no significant change in subgroups (Fig 2E).

### 3. Age-specific trends of HIV/HSV-2/syphilis seropositive rate among MSM in Shenzhen, China (2012–2018)

**Fig 3:**
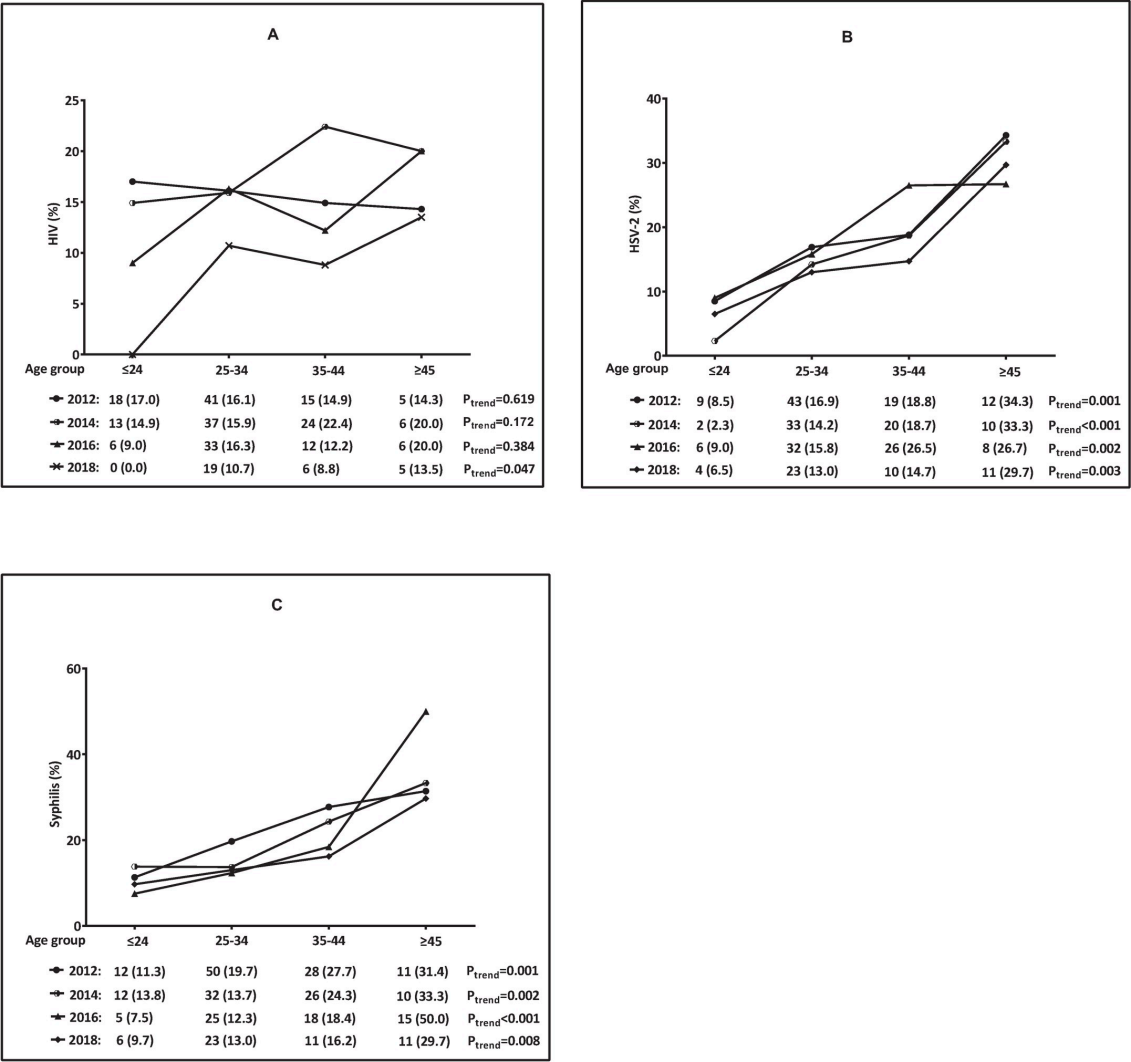
Age-specific trends of HIV/HSV-2/syphilis seropositive rate among MSM in Shenzhen, China (2012–2018). (A) Age-specific trend of HIV seropositive rate among MSM in 2012, 2014, 2016, 2018. (B) Age-specific trend of HSV-2 seropositive rate among MSM in 2012, 2014, 2016, 2018. (C) Age-specific trend of syphilis seropositive rate among MSM in 2012, 2014, 2016, 2018.

Except for 2018, we recorded no significant change with age in the HIV seropositive rate in 2012, 2014 and 2016. (2018: 0.0% in≤24 years group to 13.5% in≥45 years group; *P*_*trend*_ = 0.047) (Fig 3A). The HSV-2 seropositive rate increased sharply with age in 2012, 2014, 2016 and 2018 (*P*_*trend*_ = 0.001, *P*_*trend*_ <0.001, *P*_*trend*_ = 0.002, *P*_*trend*_ = 0.003, respectively), and the highest rate was observed in≥45 years group, about 30.0% (Fig 3B). We also observed the seropositive rate of syphilis increased significantly with age in 2012, 2014, 2016, 2018 (*P*_*trend*_ = 0.001, *P*_*trend*_ = 0.002, *P*_*trend*_ <0.001, *P*_*trend*_ = 0.008, respectively), and the highest rate was seen in the oldest group (Fig 3C).

### 4. Factors correlated with HSV-2 infection among MSM in Shenzhen, China

In univariable logistic regression analysis, the variables significantly correlated with HSV-2 infection were age group, education level, monthly income (RMB), marital status, ever had sex with female, gender of first sexual partner, number of male sex partners in P6M, frequency of condom use in anal sex with men in P6M, history of STDs (Table 2).

**Table 2:** Univariate analysis of HSV-2 infection factors among MSM in Shenzhen, China (N = 1695).

Variable	HSV-2 infected	Univariate analysis	
	n (%)	COR (95% CI)	*P*_value_
**Age group**			
≤24	21/322 (6.5)	Ref	
25–34	131/867 (15.1)	**2.55 (1.58, 4.12)**	**<0.001**
35–44	75/374 (20.1)	**3.60 (2.16, 5.99)**	**<0.001**
≥45	41/132 (31.1)	**6.46 (3.63, 11.49)**	**<0.001**
**Residence permit**			
Shenzhen	39/297 (13.1)	Ref	
Other cities of Guangdong Province	38/293 (13.0)	0.99 (0.61, 1.59)	0.953
Other Provinces	191/1105 (17.3)	1.38 (0.95, 2.00)	0.087
**Residence in Shenzhen (years)**			
0	23/107 (21.5)	Ref	
<1	46/290 (15.9)	0.69 (0.39, 1.20)	0.190
1–2	44/278 (15.8)	0.69 (0.39, 1.21)	0.190
>2	155/1020 (15.2)	0.65 (0.40, 1.07)	0.091
**Education**			
Middle school or less	87/344 (25.3)	**2.48 (1.80, 3.43)**	**<0.001**
High school	83/534 (15.5)	1.35 (0.99, 1.85)	0.062
College and above	98/817 (12.0)	Ref	
**Employment**			
Full-time employed	220/1450 (15.2)	Ref	
Part-time /self-employed	19/104 (18.3)	1.25 (0.75, 2.10)	0.399
Unemployed/retired/student/other	29/141 (20.6)	1.45 (0.94, 2.23)	0.094
**Monthly income (RMB)**			
≤3000	78/392 (19.9)	**1.69 (1.20, 2.38)**	**0.003**
3001–5000	113/703 (16.1)	1.30 (0.95, 1.78)	0.099
>5000	77/600 (12.8)	Ref	
**Marital status**			
Unmarried	179/1282 (14.0)	Ref	
Married/cohabiting	55/285 (19.3)	**1.47 (1.06, 2.06)**	**0.023**
Widowed/ separated /divorced	34/128 (26.6)	**2.23 (1.46, 3.40)**	**<0.001**
**Sexual orientation**			
Homosexual	182/1189 (15.3)	Ref	
Bisexual	63/373 (16.9)	1.12 (0.82, 1.54)	0.463
Heterosexual or unsure	23/133 (17.3)	1.16 (0.72, 1.86)	0.549
**Age at sex debut**			
≤20	94/649 (14.5)	Ref	
>20	174/1046 (16.6)	1.18 (0.90, 1.55)	0.238
**Ever had sex with female**			
No	123/934 (13.2)	Ref	
Yes	145/761 (19.1)	**1.55 (1.19, 2.02)**	**0.001**
**Gender of first sexual partner**			
Male	160/1184 (13.5)	Ref	
Female	108/511 (21.1)	**1.72 (1.31, 2.25)**	**<0.001**
**Gender of sexual partner in P6M**			
Male	231/1517 (15.2)	Ref	
Male and female	37/178 (20.8)	1.46 (0.99, 2.15)	0.056
**Number of male sex partners in P6M**			
1	56/414 (13.5)	Ref	
2–4	113/731 (15.5)	1.17 (0.83, 1.65)	0.376
≥5	61/318 (19.2)	**1.52 (1.02, 2.26)**	**0.039**
**Frequency of condom use in anal sex with men in P6M**			
Never	73/390 (18.7)	Ref	
Sometimes (<50%)	45/262 (17.2)	0.90 (0.60, 1.36)	0.616
Always (>50%)	38/253 (15.0)	0.77 (0.50, 1.18)	0.226
Every time	77/561 (13.7)	**0.69 (0.49, 0.98)**	**0.038**
**Had STD related symptoms in the past year**^**※**^			
No	217/1417 (15.3)	Ref	
Yes	51/278 (18.3)	1.24 (0.89, 1.74)	0.206
**History of STDs** ^ψ^			
No	229/1521 (15.1)	Ref	
Yes	39/174 (22.4)	**1.63 (1.11, 2.39)**	**0.012**
**Syphilis infection**			
No	171/1400 (12.2)	Ref	
Yes	97/295 (32.9)	**3.52 (2.63, 4.71)**	**<0.001**
**HIV infection**			
No	198/1449 (13.7)	Ref	
Yes	70/246 (28.5)	**2.51 (1.83, 3.44)**	**<0.001**

NOTE.

**Abbreviations:** HSV-2, Herpes simplex virus type-2; MSM, men who have sex with men; COR, crude odds ratio; CI, confidence interval; Ref, reference group; STDs, sexually transmitted diseases; RMB, Renminbi; P6M, in the past 6 months.

^**※**^ ‘symptoms’ here including at least one of the following: Painful urination or burning sensation; Abnormal of urethral discharge, Genital ulcers or warts, Anal ulcers or secretions, etc.

^ψ^ ‘STDs’ here including at least one of the following: Condyloma acuminate, Gonorrhea, urethritis, Chlamydial infection, Hepatitis B, etc. (except for HIV/syphilis infection).

Statistically significant variables (*P* <0.05 in univariate analysis) are shown in boldface.

In multicollinearity analysis, the maximum conditional index was 20.92 (>10), and the variance proportion (X4 = 0.56, X7 = 0.68, X9 = 0.99) was over 0.5. Therefore, it is showed that there was obvious multicollinearity between variables (S1 Table). The principal component analysis (PCA) extracted 4 factors: FAC1_1 (X_1_ = Ever had sex with female, X_2_ = Gender of fist sexual partner, X_3_ = Marital status, X_4_ = Age group), FAC2_1 (X_5_ = Education, X_6_ = Monthly income (RMB), X_7_ = Frequency of condom use in anal sex with men in P6M), FAC3_1 (X_8_ = Number of male sex partners in P6M), FAC4_1 (X_9_ = History of STDs) (S2 and S3 Tables). These four factors were included in principal component logistic regression analysis, and the results revealed that FAC1_1, FAC2_1 and FAC4_1 were significantly related to HSV-2 infection (Table 3).

**Table 3:** The result of principal component logistic regression analysis.

Factor ^β^	Variable	Multivariate analysis	
		AOR (95% CI)	*P*_value_
**FAC1_1**		**1.53 (1.35, 1.74)**	**<0.001**
	X_1_ = Have you ever had sex with women		
	X_2_ = Gender of first sexual partner		
	X_3_ = Marital status		
	X_4_ = Age		
**FAC2_1**		**0.85 (0.74, 0.96)**	**0.012**
	X_5_ = Education		
	X_6_ = Monthly income (RMB)		
	X_7_ = Frequency of condom use in anal sex with men in P6M		
**FAC3_1**		1.12 (0.98, 1.28)	0.094
	X_8_ = Number of male sex partners in P6M		
**FAC4_1**		**1.14 (1.01, 1.28)**	**0.034**
	X_9_ = History of STDs ^ψ^		

NOTE.

**Abbreviations:** AOR, adjusted odds ratio; CI, confidence interval; RMB, Renminbi; P6M, in the past 6 months; STDs, sexually transmitted diseases.

^β^ ‘Factor’: four principal components (FAC1_1, FAC2_1, FAC3_1, FAC4_1).

^ψ^ ‘STDs’ here including at least one of the following: Condyloma acuminate, gonorrhea, urethritis, Chlamydial infection, Hepatitis B, etc. (except for HIV/syphilis infection).

Statistic significant variables (*P* <0.05 in multiple analysis) are shown in boldface.

## Discussion

Our study observed the seropositive rate of HIV and syphilis decreased significantly among MSM in Shenzhen, China. A survey conducted in Xi’an found no significant change in HIV prevalence from 2013 to 2016, while the prevalence of syphilis showed a downward trend [16]. A serial cross-sectional survey from 2011 to 2018 in Chongqing showed that the HIV and syphilis prevalence ranged from 13.5% to 16.4%, 9.7% to 3.5%, respectively [17]. In addition, we also observed the HIV seropositive rate decreased significantly in subgroups (syphilis uninfected MSM, HSV-2 uninfected MSM, HSV-2 or syphilis uninfected MSM, HSV-2 and syphilis uninfected MSM). Many factors might contribute to the reduction in the prevalence of HIV among MSM in Shenzhen. Firstly, previous studies found that higher monthly income, fewer sexual partner, continuous use of condoms were protective factors for HIV infection [18–20]. Our result showed a significant increase from 18.3% to 60.5% in MSM proportion whose monthly income was over 5000 RMB. Furthermore, in the past six months, the proportion of one male sexual partner increased from 17.9% to 31.4%, and persistent condom use in anal sex increased from 10.9% to 50.3%. These changes in sexual behavior imply that HIV education and intervention and condom distribution efforts in MSM venues had a beneficial effect on HIV prevention among MSM. Secondly, our study found that the syphilis seropositive rate decreased significantly in MSM. Substantial epidemiological evidence has been supported that syphilis is a biological risk factor for HIV infection [21,22]. Syphilis referral and treatment program had been implemented in Shenzhen MSM from 2008. In addition, since 2010, the Chinese Ministry of Health implemented a 10-year syphilis prevention plan to control this curable disease, contributing to the decrease of syphilis among high-risk groups.

Although HIV and syphilis infections have declined, the situation remains severe. In this study, the HIV seropositive rate was 8.7% in 2018, higher than MSM in some other China regions, such as Shandong (4.4%) [23], Xi’an (7.0%) [16], Guangzhou (7.0%) [24].The seropositive rate of syphilis was 14.8% in 2018, higher than studies among MSM conducted in some other Chinese cities [23,25]. It is necessary to continue strengthening HIV and syphilis interventions among MSM in Shenzhen.

In our study, HSV-2 seropositive rate had no significant change and maintained a high level. A cross-sectional survey conducted in six Chinese cities showed that the prevalence of HSV-2 among MSM was 12.1%, which was lower than our result [8]. A survey conducted among MSM in San Francisco showed that the prevalence of HSV-2 was 26.1%, which was significantly higher than our result [26]. A multisite cross-sectional study in 3 cities in China found that the prevalence of HSV-2 in MSM was 16.0%, which was similar to our result [7]. HSV-2 is a biological risk factor for HIV infection, which can increase the risk of acquiring and transmitting HIV, even promote the progression of HIV disease. A meta-analysis reported that HSV-2 prevalence could be seen as a helpful proxy “biomarker” to track the risk of sexual behavior and forecast HIV epidemic potential. HSV-2 prevalence higher than 20% means the risk behavior was sufficient to sustain the HIV epidemic [15]. Moreover, we found that the seropositive of HSV-2 among HIV infected MSM was higher than HIV uninfected MSM. A survey in Toronto found that the prevalence of HSV-2 among HIV-positive MSM was higher than HIV-negative MSM (55.9% vs 38.2%) [9]. A cross-sectional study in Northeast China showed that the HSV-2 mono-infection rate among HIV-positive MSM was 48.6% [27]. Therefore, it is urgently to carry out HSV-2 detection and intervention among MSM in Shenzhen, especially for HIV and HSV-2 coinfection MSM.

This study showed that the seropositive rates of HIV, syphilis and HSV-2 increased significantly with age. A meta-analysis of HIV prevalence among MSM in China showed that the HIV infection rate significantly increased with age, and the HIV infection rate was the highest (19.3%) among MSM over 50 years old. The possible reason was that the elderly MSM had been exposed to HIV for a longer time, more likely to have unprotected anal sex and have a lower education level than the young MSM [28]. In our study, the HIV, syphilis, HSV-2 seropositive rate among MSM over 45 years old was about 20%, 30% and 30%. It is might due to the elderly MSM had been exposed to risk factors for a longer time. Furthermore, since HSV-2 seropositive reveals lifetime exposure to the infection, the age-specific of HSV-2 may be due to the cumulative effect of the HSV-2 antibody. A meta-analysis reported that the age-specific of HSV-2 might help identify the high-risk subgroup of HSV-2 infection, predict the HIV susceptible population, and optimize the prevention and control strategies of HSV-2 and HIV [29]. These results emphasize that HIV, HSV-2 and syphilis prevention strategies need to target different age groups, especially older MSM.

In principal component multivariate logistic analysis, FAC1_1 (X_1_ = Ever had sex with female, X_2_ = Gender of first sexual partner, X_3_ = Marital status, X_4_ = Age group) mainly reflects heterosexual behavior. With the increase of age, MSM forced by family pressure will choose to marry and have children. Therefore, the variable (age group) was included as a principal component along with X_1_, X_2_, and X_3_. Our result might indicate that MSM with heterosexual behavior was associated with HSV-2 infection (AOR = 1.53). Meanwhile, 22.0% of MSM were self-reported as bisexual in our study. Therefore, it is suggested that strengthen the HSV-2 detection and intervention in bisexual MSM. FAC2_1 (X_5_ = Education, X_6_ = Monthly income (RMB), X_7_ = Frequency of condom use in anal sex with men in P6M) mainly represents the behavior of protection, which was associated with HSV-2 infection (AOR = 0.85). Due to MSM with higher education level and income, it is more likely to acquire and accept STD-related knowledge and have the financial ability to take better protection measure (e.g. using condoms). A study in HSV-2-positive MSM found that subclinical HSV-2 shedding often occurs in the anal site [30]. Another study reported that HIV/HSV-2 co-infected MSM are frequently unaware of genital ulcers, and it is considered that the use of condoms is significant for the HSV-2 prevention strategies [13]. In this study, although the proportion of continuous condom use in anal sex increased significantly, it only accounted for 50.3% in 2018. Therefore, improving HSV-2 related knowledge and the frequency of condom use is an effective method to prevent and control HSV-2 infection and transmission among MSM in Shenzhen. Most studies demonstrated that having multiple sex partners is associated with HSV-2 infection [31,32]. Although FAC3_1 (X_8_ = Number of male sex partners in P6M) was not associated with HSV-2 infection in this study, it is necessary to prevent and control HSV-2 infection in MSM with multiple sexual partners. FAC4_1 (X_9_ = History of STDs) was associated with HSV-2 infection (AOR = 1.14), consistent with a study in western Kenya [33]. These findings were valuable for designing HSV-2 prevention and control among MSM in Shenzhen, China.

This study is the first large sample size that analyzes the trend of HSV-2 seropositive rate and factors associated with HSV-2 infection among MSM in Shenzhen, China. In contrast, our study also has several limitations. In this cross-sectional study, the relationship between HSV-2 and HIV cannot be inferred. The sample size of four years is inconsistent, which may affect the research results. All participants were recruited from MSM-frequented venues, and the findings may not be generalizable to the general MSM population in Shenzhen or other cities in China. The sexual behaviors are self-reported, resulting in an under-estimation of the actual behaviors due to social desirability bias and recall bias. The HSV-2 positive specimens did not use western blot (WB) assay to confirm the infection, which might overestimate the seropositive rate of HSV-2.

## Conclusions

In summary, the seropositive rates of HIV and syphilis have dropped significantly but are still high. HSV-2 seropositive rate had no significant change and maintained a high level. Moreover, the seropositive rates of HIV, syphilis and HSV-2 increased significantly with age. The design and delivery of HSV-2 detection and intervention among MSM in Shenzhen should focus on age, heterosexual behavior, education level and income, condom use in anal sex, multiple sexual partners, and STDs history.

## Supporting information

S1 Table Calculation results of eigenvalue analysis (Multicollinearity analysis^α^). (PDF)

S2 Table Eigenvalue, variance percentage and contribution rate of principal component (Principal component analysis). (PDF)

S3 Table The result of component matrix. (PDF)

S1 Dataset (XLS)
