# Supplementary material for: Trends of HIV/Syphilis/HSV-2 seropositive rate and factors associated with HSV-2 infection in men who have sex with men in Shenzhen, China: A retrospective study
Source: PLoS One. 2021 May 20;16(5):e0251929. doi: 10.1371/journal.pone.0251929 (PMC8136746; doi:10.1371/journal.pone.0251929)
Supplement: S1 Table — (PDF) [file pone.0251929.s001.pdf]

S1 Table. Calculation results of eigenvalue analysis (Multicollinearity analysis$^{\alpha}$)

Code	Eigenvalue	Condition Index	Variance Decomposition Proportion									
			Constant	X_1	X_2	X_3	X_4	X_5	X_6	X_7	X_8	X_9
1	7.83	1.00	0.00	0.00	0.00	0.00	0.00	0.00	0.00	0.00	0.00	0.00
2	0.91	2.94	0.00	0.00	0.00	0.00	0.00	0.00	0.00	0.00	0.00	**0.99**
3	0.57	3.70	0.00	0.30	0.00	0.00	0.00	0.01	0.01	0.02	0.01	0.00
4	0.18	6.53	0.00	0.12	0.00	0.16	0.09	0.00	0.00	0.26	0.14	0.00
5	0.17	6.77	0.00	0.07	0.00	0.09	0.04	0.12	0.12	0.01	0.24	0.00
6	0.12	8.02	0.00	0.01	0.00	0.06	0.01	0.03	0.00	**0.68**	0.41	0.00
7	0.08	9.93	0.00	0.00	0.00	0.48	**0.56**	0.12	0.09	0.00	0.00	0.00
8	0.07	**10.84**	0.03	0.13	0.33	0.17	0.01	0.02	0.37	0.00	0.11	0.00
9	0.06	**11.97**	0.00	0.17	0.23	0.02	0.24	0.45	0.39	0.03	0.00	0.00
10	0.02	**20.92**	**0.96**	0.21	0.44	0.02	0.06	0.24	0.02	0.01	0.09	0.00

NOTE.

$^{\alpha}$: Dependent variable=HSV-2 infection.

X_1=Ever had sex with female; X_2=Gender of first sexual partner; X_3=Marital status; X_4=Age group; X_5=Education; X_6=Monthly income (RMB); X_7=Frequency of condom use in anal sex with men in P6M; X_8=Number of male sex partners in P6M; X_9=History of STDs$^{\Psi}$.

Abbreviations: RMB, Renminbi; P6M, in the past 6 months; STDs, sexually transmitted diseases.

$^{\Psi}$'STDs' here including at least one of the following: Condyloma acuminate, Gonorrhea, urethritis, Chlamydial infection, Hepatitis B, etc. (except for HIV/syphilis infection).

Condition Index ≥10 and Variance Decomposition Proportion >0.5 are shown in boldface.
