# Supplementary material for: Trends of HIV/Syphilis/HSV-2 seropositive rate and factors associated with HSV-2 infection in men who have sex with men in Shenzhen, China: A retrospective study
Source: PLoS One. 2021 May 20;16(5):e0251929. doi: 10.1371/journal.pone.0251929 (PMC8136746; doi:10.1371/journal.pone.0251929)
Supplement: S2 Table — (PDF) [file pone.0251929.s002.pdf]

S2 Table. Eigenvalue, variance percentage and contribution rate of principal component (Principal Component Analysis)

Component	Initial Eigenvalue			Extraction sums of squared Loads		
	Total	% of Variance	Cumulative %	Total	% of Variance	Cumulative %
1	2.43	27.00	27.00	2.43	27.00	27.00
2	1.43	15.84	42.85	1.43	15.84	42.85
3	1.23	13.61	56.46	1.23	13.61	56.46
4	1.00	11.16	67.62	1.00	11.16	67.62
5	0.91	10.09	77.70			
6	0.69	7.71	85.41			
7	0.58	6.46	91.86			
8	0.46	5.16	97.02			
9	0.27	2.98	100			
