# Supplementary material for: Trends of HIV/Syphilis/HSV-2 seropositive rate and factors associated with HSV-2 infection in men who have sex with men in Shenzhen, China: A retrospective study
Source: PLoS One. 2021 May 20;16(5):e0251929. doi: 10.1371/journal.pone.0251929 (PMC8136746; doi:10.1371/journal.pone.0251929)
Supplement: S3 Table — (PDF) [file pone.0251929.s003.pdf]

S3 Table. The result of component matrix

Factor [β]	Variable	Component			
		1	2	3	4
**FAC1_1**	X_1=Ever had sex with female	**0.81**	0.19	-0.09	0.00
	X_2=Gender of first sexual partner	**0.80**	0.11	-0.05	-0.06
	X_3=Marital status	**0.72**	0.12	-0.08	-0.03
	X_4=Age group	**0.62**	0.18	-0.05	0.02
**FAC2_1**	X_5=Education	-0.44	**0.58**	-0.38	-0.03
	X_6=Monthly income (RMB)	-0.18	**0.79**	-0.29	0.07
	X_7=Frequency of condom use in anal sex with men in P6M	-0.07	**0.58**	0.56	-0.08
**FAC3_1**	X_8=Number of male sex partners in P6M	0.02	0.20	**0.81**	0.03
**FAC4_1**	X_9=History of STDs [Ψ]	0.05	0.01	0.03	**0.99**

NOTE.

Abbreviations: RMB, Renminbi; P6M, in the past 6 months; STDs, sexually transmitted diseases.

[β] 'Factor': four principal components (FAC1_1, FAC2_1, FAC3_1, FAC4_1) and are shown in boldface.

[Ψ]'STDs' here including at least one of the following: Condyloma acuminate, gonorrhea, urethritis, Chlamydial infection, Hepatitis B, etc. (except for HIV/syphilis infection).
